# The Effect of Polyunsaturated Aldehydes on *Skeletonema marinoi* (Bacillariophyceae): The Involvement of Reactive Oxygen Species and Nitric Oxide

**DOI:** 10.3390/md12074165

**Published:** 2014-07-14

**Authors:** Alessandra A. Gallina, Christophe Brunet, Anna Palumbo, Raffaella Casotti

**Affiliations:** Stazione Zoologica Anton Dohrn di Napoli, Villa Comunale, Napoli I80121, Italy; E-Mails: christophe.brunet@szn.it (C.B.); palumbo@szn.it (A.P.)

**Keywords:** octadienal, heptadienal, infochemical, xanthophylls, signaling, competition

## Abstract

Nitric oxide (NO) and reactive oxygen species (ROS) production was investigated in the marine diatom, *Skeletonema marinoi* (SM), exposed to 2*E*,4*E*/*Z*-decadienal (DECA), 2*E*,4*E*/*Z*-octadienal (OCTA), 2*E*,4*E*/*Z*-heptadienal (HEPTA) and a mix of these last two (MIX). When exposed to polyunsaturated aldehydes (PUA), a decrease of NO was observed, proportional to the PUA concentration (85% of the initial level after 180 min with 66 µM DECA). Only OCTA, HEPTA and MIX induced a parallel increase of ROS, the highest (2.9-times the control) with OCTA concentrations twice the EC_50_ for growth at 24 h (20 μM). The synthesis of carotenoids belonging to the xanthophyll cycle (XC) was enhanced during exposure, suggesting their antioxidant activity. Our data provide evidence that specific pathways exist as a reaction to PUA and that they depend upon the PUA used and/or the diatom species. In fact, *Phaeodactylum tricornutum* (PT) produces NO in response to DECA, but not to OCTA. We advance the hypothesis that SM perceives OCTA and HEPTA as intra-population infochemicals (as it produces PUA), while PT (non-PUA producing species) perceives them as allelochemicals. The ability to produce and to use PUA as infochemicals may underlie ecological traits of different diatom species and modulate ecological success in natural communities.

## 1. Introduction

Marine diatoms are one of the most successful groups of eukaryotic photosynthetic organisms on Earth and are major players in global marine primary production by representing most of the organic carbon that is at the base of the marine food web [1]. About 30% of the marine diatoms tested so far have been found to produce polyunsaturated aldehydes (PUA) as secondary metabolites derived from fatty acid metabolism [2]. PUA, together with a plethora of other different metabolites derived from the same biosynthetic pathway, all belonging to oxylipins, play key roles in chemically-mediated plankton interactions (for a review, see [3]).

Since their first identification in marine diatoms [4], PUA have been demonstrated to have negative effects on copepod development and reproduction, as well as on other marine invertebrates [5,6,7,8]. PUA do not act only as a chemical defense against grazers, but also as signals, determining the fate of the pelagic microbial community structure. Indeed, PUA affect bacterial community composition, with some groups being more sensitive and others highly resistant [9,10]. They also inhibit the growth of phytoplankton species belonging to different taxonomical groups, thereby possibly acting as allelochemicals [11,12]. Thus, it is speculated that a stress surveillance system might exist in diatoms in response to PUA, which leads to population-level cell death and bloom termination at specific PUA concentrations [13]. In the diatom, *Phaeodactylum tricornutum*, this stress-surveillance system induces the production of nitric oxide (NO) and a transient rise in intracellular Ca^2+^ that later activates a gene cascade involved in programmed cell death [13,14].

NO is a versatile molecule involved in many different processes in animals, such as neurotransmission, vasodilatation and defense against pathogens (e.g., [15,16]). In plants, NO regulates a number of different genes involved, for instance, in photosynthesis, cell death and basic cellular metabolism (for a review, see [17]). Compared to animals and higher plants, studies of NO production in marine phytoplankton are limited. NO is reported to regulate physiological processes and stress responses in the chlorophycean, *Scenedesmus obliquus* [18,19], and ichthyotoxicity in raphidophycean flagellates [20]. In addition, NO production by the dinoflagellate, *Symbiodinium* spp., occurs during coral bleaching [21,22]. NO is also involved in sensing PUA-derived stress [13], in regulating gene expression and subsequent cell death under light stress [23], in controlling the adhesion of benthic diatoms to different substrates [24] and in inhibiting biofilm formation in response to PUA [25]. In addition, NO is produced during normal growth in different phytoplankton species [26,27], suggesting that it can function as a growth-regulating factor.

Apart from NO, other molecules are involved in the stress response of different organisms. Reactive oxygen species (ROS), such as superoxide radical (O_2_^−^), hydrogen peroxide (H_2_O_2_) and singlet oxygen (^1^O_2_), are natural byproducts of cell metabolism and are commonly produced in response to different stress agents, including heat, UV radiation, chemicals and, more generally, to any stressful change in environmental conditions [28]. In phytoplankton, CO_2_ limitation [29], viral infection [30], high pH and iron limitation [31], prolonged darkness [32], as well as cadmium [33] and paraquat exposure [34] are reported to induce an increase in ROS. Furthermore, compounds involved in biotic interactions are associated with ROS production, such as thiol proteases excreted by ageing cells [35], the cyanobacterial toxin nodularin [36] and other macrophytes-derived allelochemicals [37].

The concomitant production of NO and ROS (hydrogen peroxide) is, indeed, reported to be involved in the wound-activated response of the macroalga, *Dasycladus vermicularis* [38]. In this case, their production is also partly co-regulated, supporting the conclusion that a signaling relationship exists between ROS and reactive nitrogen species (RNS), as previously suggested in algae and plants [39,40].

NO and ROS are toxic and reactive molecules causing strong damage to many cell components. To cope with the related oxidative stress and to prevent or limit irreversible damages, cells have developed several mechanisms. Among the observed responses, there is the increase of the two antioxidative enzymes, superoxide dismutase (SOD) and glutathione peroxidase (GPx) [36], and the accumulation of carotenoids [41]. In the freshwater *Haematococcus pluvialis*, the carotenoid, astaxanthin, is accumulated during the encystment process triggered by paraquat exposure, with a negative linear relationship with ROS [34]. In the marine diatom, *Thalassiosira weissflogii* (TW), photoprotective xanthophyll carotenoid pigments increase when cells are exposed to PUA, suggesting that they play a role as antioxidants independently of light [11].

The general aim of this study is to investigate the responses of the diatom, *Skeletonema marinoi* (SM), to PUA in terms of NO and ROS production, to test the hypothesis that these responses underlie an adaptive advantage leading to ecological success. Opposite from *Thalassiosira weissflogii* (TW) [11] and *Phaeodactylum tricornutum* (PT) [13], which do not produce PUA [2], SM produces high amounts of PUA, mainly 2*E*,4*E*/*Z*-octadienal, 2*E*,4*E*/*Z*-heptadienal and 2*E*,4*E*/*Z*,7-octatrienal [42]. Moreover, SM is a successful species in nature, being widespread worldwide and forming spring or autumn blooms in many coastal areas [43]. Our study focuses on NO and ROS production (and their interrelationship) in response to PUA and on the activation of the xanthophyll cycle (XC) as a protection mechanism to maintain photosynthetic performance. The differences between PT and SM in terms of NO production are analyzed comparatively to infer their involvement in the ecological success of SM and interpreted as an adaptive response of diatoms to chemical cues in general.

## 2. Results

### 2.1. Nitric Oxide Production

SM cultures were inoculated with different concentrations of three PUA (2*E*,4*E*/*Z*-decadienal (DECA), 2*E*,4*E*/*Z*-octadienal (OCTA), 2*E*,4*E*/*Z*-heptadienal (HEPTA)) or a 1:1.4 mix of OCTA and HEPTA (MIX). Values were normalized by the values of the controls measured at the same time. Controls are samples treated exactly like the others, except for the PUA (zero-PUA). The normalization is necessary in order to avoid the natural variability in physiological NO levels hiding the new NO production. DECA has been chosen because it is a commonly used PUA in toxicity tests on several model and non-model organisms (e.g., [25,44]). OCTA and HEPTA are, instead, the PUA commonly produced by SM, as well as by other diatoms [2]. The concentrations used were multiples of the EC_50_ concentration for growth at 24 h for SM, as reported in [12] (*i.e.*, 2.48 μmol·L^−1^ for DECA, 8.94 μmol·L^−1^ for OCTA and 18.17 μmol·L^−1^ for HEPTA), and the concentrations reported to elicit NO production in PT (33 μM and 66 μM DECA) [13]. The mix of OCTA and HEPTA reflected the ratio of these two PUA in SM cultures, as reported by [42].

**Figure 1:**
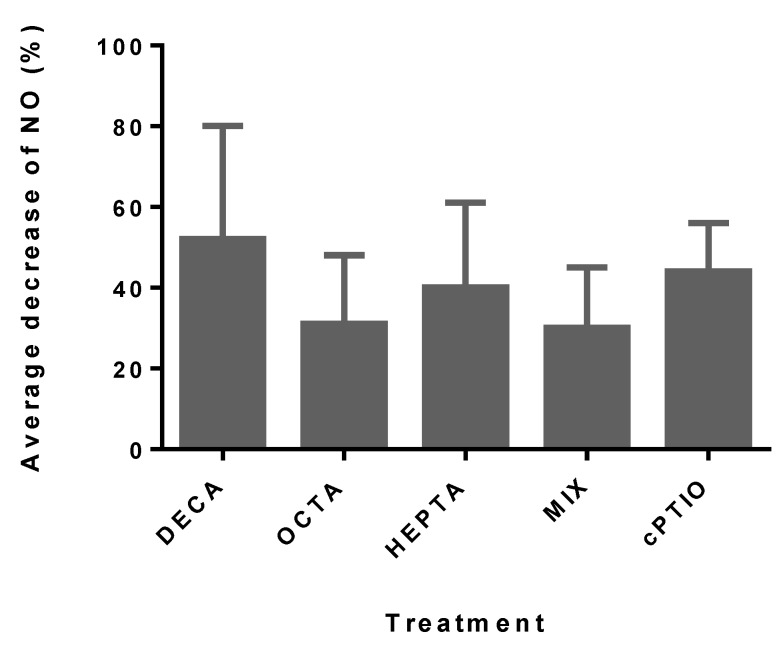
The average decrease of NO in *Skeletonema marinoi* (SM) expressed as the percent (%) of decrease relative to the control (zero-PUA, 100%) after 180 min of exposure to the different PUA treatments (the average of all concentrations tested). Data are the means ± SD, *n* = 6. cPTIO is a NO scavenger (carboxy-PTIO, negative control), inoculated at the same concentration as the highest concentration used for the PUA tested (see Table 1). DECA, 2*E*,4*E*/*Z*-decadienal; OCTA, 2*E*,4*E*/*Z*-octadienal; HEPTA, 2*E*,4*E*/*Z*-heptadienal; MIX, mix of OCTA and HEPTA.

When exposed to DECA, no increase in 4-amino-5-methylamino-2′,7′-difluorofluorescein diacetate (DAF-FM DA)-green fluorescence was observed, indicating the absence of NO production. Rather, a NO decrease was observed after 180 min of exposure with all the concentrations tested (Table 1). The decrease, although less striking, was also observed in cultures exposed to the other PUA (Figure 1 and Table 1), and it was also due to a consumption of the basal levels of NO, as also confirmed by the comparable effects obtained with the NO scavenger carboxy-PTIO, (cPTIO) (Figure 1). The average decrease of NO after 180 min was 52% (SD ± 28%) with DECA, 31% (SD ± 17%) with OCTA and 40% (SD ± 21%) with HEPTA (Figure 1). After 180 min, fluorescence values were significantly different than the controls and, among them, for all the DECA concentrations tested (Table 1). With OCTA, a significant difference in NO relative to the control was evident starting from OCTA concentrations of 1 µM (Table 1), while for HEPTA, NO reduction at 180 min started to be significant at 5 µM. When SM was exposed to the MIX, no synergistic effect was observed, as the NO values were similar to the ones elicited by OCTA alone (30%, SD ± 15%) (Figure 1), and a significant reduction in NO was evident only with 10 µM OCTA + 14 µM HEPTA and 20 µM OCTA + 28 µM HEPTA.

**Figure 2:**
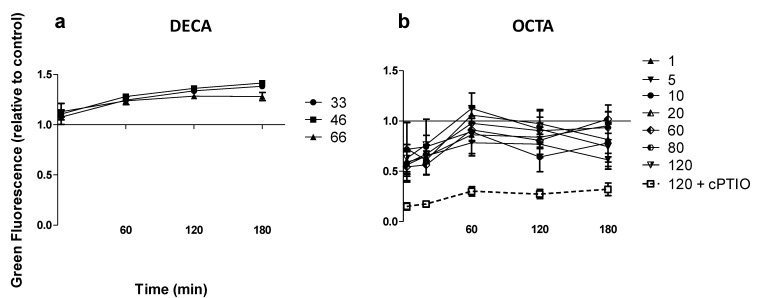
NO production in *Phaeodactylum tricornutum* (PT) exposed to DECA and OCTA. (**a**) DECA; (**b**) OCTA. Concentrations in the legends are expressed in μM. Data are fluorescence values normalized to the control value (zero PUA, equal to one) and are the means ± SD, *n* = 3 (biological replicates). cPTIO was used as an NO scavenger (negative control) inoculated at the same concentration as the highest concentration used for the PUA tested in each experiment.

Since NO was previously reported to be produced by the diatom, PT, in response to DECA [13], we tested NO production in PT upon exposure to DECA, in order to exclude an artifact in our experiments, due, for instance, to different culture conditions or methodological procedures. NO production in PT upon exposure to DECA was indeed confirmed (Figure 2a). When PT was exposed to OCTA, instead (not tested in [13]), NO was not produced (Figure 2b), and a decreasing gradient of DAF-FM-NO fluorescence was observed, similar to SM, showing, indeed, a differential response to different PUA in PT. The NO scavenger, cPTIO (negative control), dramatically reduced green fluorescence from DAF-FM-NO, further supporting this interpretation.

**Table 1:** The significance of the NO decrease after 180 min of exposure to the different PUA. cPTIO was used as an NO scavenger (negative control) inoculated at the same concentration as the highest concentration used for the PUA tested in each experiment. The MIX is a 1:1.4 mix of OCTA and HEPTA, and the concentration indicated is the one of the OCTA. The significance is calculated using the Student’s *t*-test; (*) *p* < 0.05; ns: not significant; *n* = 3.

Concentration (μM)	DECA	OCTA	HEPTA	MIX
0.05	*	ns	ns	ns
0.1	*	ns	ns	ns
1	*	*	ns	ns
5	*	*	*	ns
10	-	*	*	*
20	-	*	*	*
33	*	-	-	-
40	-	-	*	-
66	*	-	-	-
cPTIO	*	*	*	*

### 2.2. ROS Production

The hypothesis that reactive species other than NO are involved in the stress response of SM to PUA was tested, using flow cytometry and the ROS-sensitive dye, dihydrorhodamine 123 (DHR).

**Figure 3:**
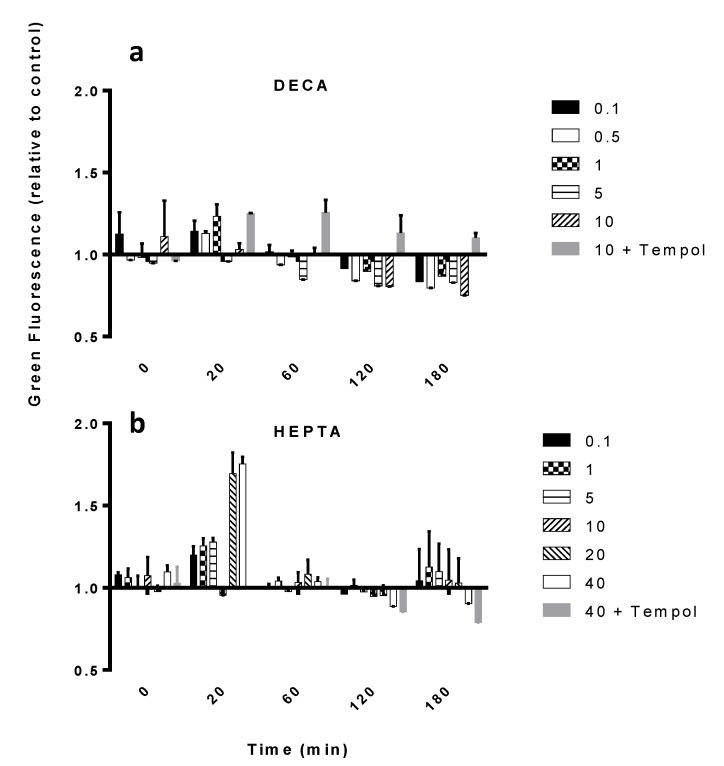
ROS production in SM exposed to different PUA. (**a**) DECA; (**b**) HEPTA. Concentrations in the legends are expressed in μM. Data are fluorescence values normalized to the control value (basal dihydrorhodamine 123 (DHR) fluorescence, zero-PUA) and are the means ± SD, *n* = 3 (biological replicates). Tempol was used as an ROS scavenger (negative control) inoculated at the same concentration as the highest concentration used for the PUA tested in each experiment.

**Figure 4:**
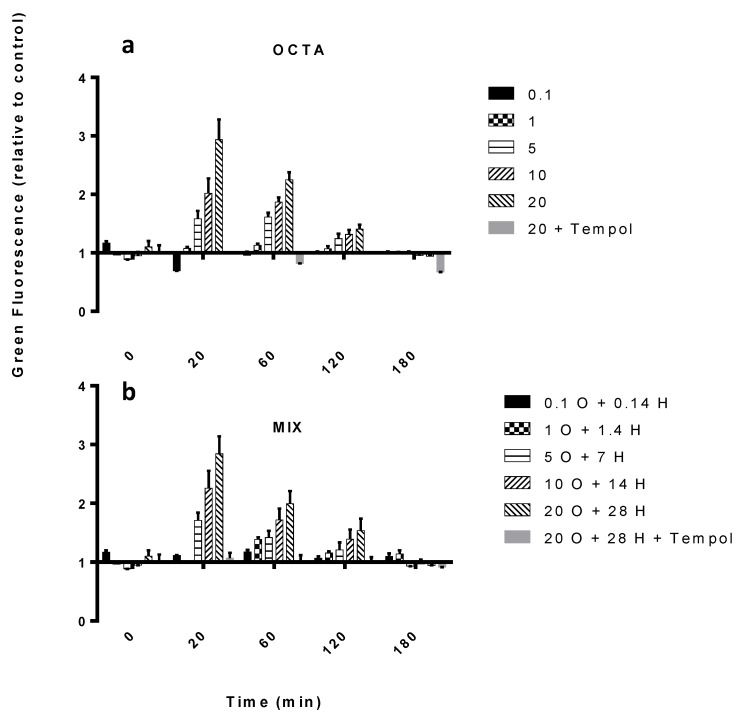
ROS production in SM exposed to different PUA. (**a**) OCTA; (**b**) MIX (O = OCTA, H = HEPTA). Concentrations in the legends are expressed in μM. Data are fluorescence values normalized to the control value (basal DHR fluorescence) and are the means ± SD, *n* = 3 (biological replicates). Tempol was used as a ROS scavenger (negative control) inoculated at the same concentration as the highest concentration used for the PUA tested in each experiment.

When exposed to DECA, SM did not show any significant increase in ROS (Figure 3a), as contrary to the other PUA and the MIX (Figure 3b and Figure 4a,b). In fact, these induced a peak 20 min after exposure to concentrations equal to or higher than 5 μM OCTA (*p* < 0.05, *n* = 3) and 20 μM HEPTA (*p* < 0.01, *n* = 3), respectively. The highest increase in ROS (2.9 times the control, *p* < 0.001, *n* = 3, Figure 4a) was induced by 20 μM OCTA. When inoculated together, OCTA and HEPTA elicited an increase in ROS comparable to OCTA alone (2.9-times the control with 20 μM OCTA plus 28 μM HEPTA, *p* < 0.001, *n* = 3, Figure 4b), therefore excluding, again, a synergistic effect of the two PUA when inoculated together. Preliminary tests included acetaldehyde as a control, in order to exclude general toxicity due to the aldehydic group. This was then excluded from further tests, as no effect was observed. In all of these experiments, cells inoculated with PUA together with the ROS scavenger, Tempol, showed a lower DHR-ROS-derived green fluorescence, confirming that the observed increase in fluorescence was really due to ROS production (Figure 3 and Figure 4).

### 2.3. Xanthophyll Cycle Activation and Photosynthetic Efficiency

The xanthophyll cycle (XC) in diatoms involves the enzymatic removal of the epoxy groups from xanthophyll diadinoxanthin (Ddx) to form the de-epoxidized xanthophyll diatoxanthin (Dtx). The XC is activated by high light conditions, playing a key role in photoprotecting the photosystems and being responsible for energy dissipation by non-photochemical quenching (NPQ), a mechanism to reduce the amount of energy that reaches the photosynthetic reaction centers. It has been already shown that the XC might be also activated by chemical stress [11].

In OCTA-exposed SM cultures, an increase in Dtx (Table 2) was observed at all the concentrations tested, after one hour of exposure, and this increase was significant for the concentrations >10 µM (*p* < 0.05, *n* = 3). Since also Ddx increased, we can conclude that this was a response of the whole xanthophyll cycle, with the cells activating the photoprotective biochemical pathway, which leads to an increase of Ddx and, consequently Dtx. Indeed, β-carotene, a xanthophyll upstream precursor of XC pigments, decreased in the first 20 min after exposure and subsequently increased up to 24 h in cultures exposed to 20 µM OCTA, indicating its involvement in the antioxidant response to PUA (Table 2). Interestingly, the de-epoxided state (DES: Dtx/(Ddx + Dtx), Table 2) started to increase after 20 min of exposure, highlighting the fast de-epoxidation activity after chemical exposure. The highest increase in Dtx and DES was observed with 20 µM OCTA after 3 h (Table 2).

**Table 2:** Xanthophyll cycle (XC) pigments (de-epoxidized xanthophyll diatoxanthin (Dtx), xanthophyll diadinoxanthin (Ddx) and the de-epoxided state (DES) = Dtx/(Ddx + Dtx)), β-carotene, non-photochemical quenching (NPQ) and growth rates (μ) in SM exposed to different concentrations of OCTA and monitored for 48 h. Data are the means ± SD, *n* = 3 (biological replicates). Values are normalized to the control, except for NPQ and the growth rates (day^−1^). Values discussed in the text are highlighted in bold.

Treatment	Time (h)	Ddx	Dtx	DES	β-carotene	NPQ	μ (day^−1^)
5 µM OCTA	0	1.07 ± 0.14	0.95 ± 0.18	0.91 ± 0.23	1.07 ± 0.05	-	-
	0.3	0.51 ± 0.09	0.79 ± 0.10	1.52 ± 0.27	**0.63 ± 0.08**	**0.95 ± 0.04**	-
	1	1.29 ± 0.11	1.51 ± 0.25	1.30 ± 0.31	1.00 ± 0.09	**0.88 ± 0.03**	-
	3	2.36 ± 0.51	1.57 ± 0.51	0.95 ± 0.49	1.30 ± 0.48	**0.88 ± 0.02**	-
	24	0.59 ± 0.07	1.20 ± 0.36	1.86 ± 0.63	**0.70 ± 0.07**	**1.10 ± 0.04**	**0.42 ± 0.08**
	48	-	-	-	-	-	−0.19 ± 0.12
10 µM OCTA	0	0.84 ± 0.03	0.89 ± 0.13	1.05 ± 0.17	1.00 ± 0.06	-	-
	0.3	0.48 ± 0.11	0.80 ± 0.15	1.61 ± 0.17	**0.61 ± 0.11**	**0.92 ± 0.03**	-
	1	1.37 ± 0.14	1.77 ± 0.04	1.50 ± 0.30	0.95 ± 0.12	**0.95 ± 0.00**	-
	3	1.21 ± 0.11	2.14 ± 1.52	1.78 ± 0.64	1.20 ± 0.30	**1.00 ± 0.03**	-
	24	1.23 ± 0.58	1.76 ± 1.17	2.37 ± 0.42	**1.08 ± 0.25**	**1.25 ± 0.04**	0.11 ± 0.15
	48	-	-	-	-	-	**−0.12 ± 0.13**
20 µM OCTA	0	0.75 ± 0.12	0.84 ± 0.07	1.12 ± 0.20	0.89 ± 0.01	-	-
	0.3	0.63 ± 0.22	1.10 ± 0.01	1.90 ± 0.46	**0.63 ± 0.16**	**0.90 ± 0.17**	-
	1	0.82 ± 0.10	2.19 ± 0.05	1.80 ± 0.37	0.87 ± 0.16	**1.01 ± 0.04**	-
	3	2.28 ± 0.46	4.51 ± 1.57	1.41 ± 0.38	1.56 ± 0.46	**1.05 ± 0.00**	-
	24	1.71 ± 0.49	**5.83 ± 1.60**	2.88 ± 0.17	**2.04 ± 0.23**	**1.32 ± 0.02**	**−0.29 ± 0.08**
	48	-	-	-	-	-	**−0.10 ± 0.10**

OCTA = 2*E*,4*E*/*Z*-octadienal; Ddx = diadinoxanthin; Dtx = diatoxanthin; NPQ = non-photochemical-quenching, μ = growth rate; DES = de-epoxidated state. In bold, values specifically discussed in the text.

Without a doubt, the activation of the XC was independent of excess light, as also confirmed by the constant NPQ values measured over the different conditions and during all of the experiment. Indeed, the NPQ value of *ca*. one has been observed within the first 3 h and throughout the 24 h of exposure (Table 2), suggesting that the activation of the XC was not involved in excess light energy dissipation, but, rather, in protecting the cells from another source of oxidative stress (*i.e.*, PUA-induced ROS production). Additionally, Fv/Fm values (where Fv is variable fluorescence and Fm maximum fluorescence), which measure photochemical efficiency of photosystem (PS) II quantum efficiency, did not vary in response to OCTA exposure and remained constantly high (*ca*. 0.66), indicating that the photosynthetic system was not being impaired by the oxidative stress induced by PUA (data not shown).

### 2.4. Growth and Recovery from PUA Stress

The growth rate after 24 h decreased from 0.42 ± 0.08 day^−1^ with 5 μM OCTA (virtually no effect compared to the control) to −0.29 ± 0.08 day^−1^ with 20 μM. After 48 h, the growth rate was as low as −0.12 ± 0.13 day^−1^ and −0.10 ± 0.10 day^−1^ with 10 µM and 20 µM (Table 2).

In order to verify the reversibility of PUA effect, OCTA-exposed cultures were resuspended in fresh medium with no OCTA added and sampled for 48 h. Twenty four hours after resuspension, the growth rate of cultures exposed to 5 µM and 10 µM had resumed to 0.69 ± 0.1 day^−1^ and 0.57 ± 0.04 day^−1^, respectively, thus comparable to that of the control (0.48 ± 0.05 day^−1^, Table 3). On the contrary, the growth rate of cultures previously exposed to the highest OCTA concentration (20 µM) increased to only 0.09 ± 0.07 day^−1^ after 24 h, but had increased to 0.47 ± 0.11 day^−1^ after 48 h.

Diatoxanthin content had decreased 24 h after resuspension for all of the OCTA concentrations (Table 3). This was particularly evident in cultures exposed to 20 µM OCTA, for which Dtx decreased 43% (from 5.83 ± 1.60-times the control 24 h after inoculation, to 3.30 ± 0.12-times the control 24 h after resuspension) (Table 2 and Table 3, respectively). Concomitantly to a decrease in Dtx, an increase in Ddx (39% increase for 20 µM OCTA) was evident, also parallel to a decrease in the DES (Table 3). β-carotene showed, instead, a different pattern, decreasing its concentration in the first 20 min of OCTA exposure at the highest OCTA concentration (Table 2) and then increasing again both during the remaining exposure time and 24 h after resuspension (Table 2 and Table 3). For the other OCTA concentrations tested (5 µM and 10 µM), β-carotene values did not vary significantly after the resuspension (Table 2 and Table 3).

Since no change from the original value of 0.66, close to the value indicated as optimal by Falkowsky and Raven [45], was detected in Fv/Fm or NPQ after 24 h of exposure, these parameters were not further measured after the resuspension of cultures in fresh medium.

**Table 3:** XC pigments Dtx, Ddx and DES = Dtx/(Ddx + Dtx), β-carotene and growth rates in SM following resuspension in fresh medium with no PUA following 24 h after exposure to different concentrations of OCTA and monitored up to 48 h. Data are the means ± SD, *n* = 3 (biological replicates). Values are normalized to the control, except for growth rates (day^−1^). Values discussed in the text are highlighted in bold.

Treatment	Time (h)	Ddx	Dtx	DES	β-carotene	μ (day^−1^)
5 µM OCTA	24	0.80 ± 0.25	0.82 ± 0.53	0.92 ± 0.38	0.88 ± 0.18	**0.69 ± 0.10**
	48	-	-	-	-	0.34 ± 0.12
10 µM OCTA	24	1.22 ± 0.76	1.20 ± 0.76	0.98 ± 0.04	0.92 ± 0.27	**0.57 ± 0.04**
	48	-	-	-	-	0.46 ± 0.08
20 µM OCTA	24	2.79 ± 0.26	**3.30 ± 0.12**	1.00 ± 0.17	2.34 ± 0.28	**0.09 ± 0.07**
	48	-	-	-	-	**0.47 ± 0.11**

OCTA = 2*E*,4*E*/*Z*-octadienal; Ddx = diadinoxanthin; Dtx = diatoxanthin; μ = growth rate; DES = de-epoxidated state. In bold, values specifically discussed in the text.

## 3. Discussion

### 3.1. Protective Responses

The fact that we could not detect an increase in NO production in SM exposed to PUA is surprising as NO appears to be involved in different stress responses in marine macro- and micro-algae [22,38,46], including diatoms [13]. In *Skeletonema costatum* (now replaced as *S. tropicum* [47]), Chung *et al.* [23] observed NO production in response to light stress, and this was also confirmed in SM [48]. This suggests that NO production in SM is stress-type dependent. Interestingly, the PUA DECA induces an increase of NO in another diatom, PT, revealing that the response to PUA is species specific and suggesting that different stress-signaling pathways are elicited by DECA in the two species. Moreover, not all PUA induce the same response, since PT produces NO only for DECA and not for the other PUA tested.

Despite being the most used PUA in toxicological experiments, DECA is not the most common PUA present in marine phytoplankton. In a survey of 51 species of marine diatoms, DECA was the least detected PUA [2], in agreement with what it has been observed at sea during diatom blooms [49]. DECA is reported *in situ* only during blooms of the prymnesiophyte, *Phaeocystis pouchetii* [50,51]. In PT, which does not produce any PUA [2], the response elicited by DECA should be attributable to its toxicity only. This reinforces the hypothesis that commonly present PUA, such as OCTA and HEPTA, act as infochemicals.

It is important to point out that not only was there no increase in NO in response to PUA exposure observed in SM, but, indeed, NO-related fluorescence decreased in both SM (exposed to all PUA) and PT (exposed to OCTA), as also confirmed by the effect of the NO scavenger. This suggests an impairment of physiological NO production, probably related to growth inhibition, which has already been shown to be an effect of PUA [11,12]. The difference in NO content in these two diatom species might be related to their different growth capabilities in culture, much higher in PT than in SM, which is confirmed by the stronger scavenging effect of cPTIO (Figure 2b).

In the Antarctic chlorophyte, *Chlorella* sp., a peak in NO marks the passage from lag to the exponential phase of growth, suggesting that NO is involved in growth regulation/modulation [26]. Additionally, a correlation is found between NO production and growth in different marine phytoplankton species, including *S. costatum*, with a peak in NO occurring right before the highest cell density [46]. This also implies that NO is normally present in the cells during growth and has a role in key passages of the physiological regulation of growth. Instead, it is hypothesized that PT might react differently to OCTA, because this PUA is believed to be more common in nature and consequently recognized as a signal. This observation calls for further studies comparing the reaction of different diatom species to PUAs and also a deeper understanding of the complex phenomena underlying the different steps involved. Among these, it cannot be excluded that intermediate chemical products are responsible for the observed effects, rather than the PUAs themselves. This does not reduce the value of the data presented, as PUAs have been identified as the main molecules inducing different effects in different model and non-model organisms [3].

The decrease in NO levels upon exposure to PUA may be due to a shift of the stress response pathway towards the production of different N or O reactive species or a plethora of them. Among these, we have chosen to investigate ROS.

Our results show that when SM is exposed to PUA other than DECA, ROS production occurs, with a peak 20 min after exposure and a threshold at half the EC_50_ for growth at 24 h for OCTA and the EC_50_ for growth at 24 h itself for HEPTA. This agrees with [13], reporting that in PT, a threshold exists in cells sensing a PUA, although its value was not quantified. The difference in the values of this threshold between OCTA and HEPTA is probably related to the stronger activity of OCTA [12], due to its relatively longer C chain.

Remarkably, DECA does not elicit ROS production in SM. This suggests that the response mediated by PUA is not simply due to the toxicity of the aldehydic group, but indeed, it is a specific reaction to specific PUA that the cells recognize as self-produced. It is therefore possible that ROS have a similar role as NO in DECA-exposed PT and that ROS (and not NO) are involved in the intra-population stress signaling pathway in SM. The ROS downstream response is likely to activate genes involved in either alternate signaling pathways or a cell death cascade, depending on the PUA concentration. In the congeneric *S. tropicum* (previously S. *costatum*) a cell death-specific gene (*ScDSP*) is involved in the cascade leading to cell death by apoptosis [52]. The expression of this gene is enhanced by the NO donor, diethylamine nitric oxide (DEANO) [23], and even more by hydrogen peroxide [53] . Additionally, in *S. tropicum*, PUA have also been shown to both induce ROS production and *ScDSP* expression, indicating a possible role of PUA in the cell death cascade in a congeneric diatom [53]. This suggests that ROS have an important role in the molecular cascade following PUA exposure, and this has strong implications for population dynamics, such as, for instance, during the later phases of diatom blooms, when cell lysis increases and PUA are released [54], allowing for a higher PUA concentration to be present. ROS production has been reported in other phytoplankton species in response to different stresses. Vardi *et al.* [35] reported that in the dinoflagellate, *Peridinium gatunense*, a thiol protease excreted by ageing cells was able to induce ROS production and concomitant cell death at the population level. Additionally, the cyanobacterial toxin, nodularin, has been found to induce an increase oxidative stress in the red alga, *Furcellaria lumbricalis* [36], and different allelochemicals produced by submerged freshwater macrophytes were reported to increase ROS production in both green algae and cyanobacteria [37].

In the field of chemical ecology, an important aspect to be taken into consideration is the ecological relevance of the concentrations used in laboratory studies and how they relate to naturally-occurring and, therefore, ecologically-relevant concentrations. In the case of PUA, only a few studies have addressed the important issue of measurements of PUA concentrations directly at sea [54,55]. In a field study in the Northern Adriatic Sea (Italy), Vidoudez *et al.* [54] reported the patchy distribution of PUA associated with a spring bloom of the diatom, *S. marinoi*, which was found to be the major contributor to the total PUA detected. Dissolved HEPTA and OCTA concentrations were found to be in the nanomolar range. Similarly, another recent study by Ribalet *et al.* [55] reported the presence of nanomolar concentrations of PUA in the Northern Adriatic Sea following diatom cell lysis. Even though these concentrations are considerably lower compared to the ones used in the present study (which are in the micromolar range), it must be considered that the methodology used to estimate PUA concentrations at sea averages values over liters of sea water, while smaller local patches of PUA at a much higher concentration are expected to be produced in the immediate surroundings of diatom cells when lysing. In fact, Ribalet *et al.* [55] argued and proposed calculations showing that concentrations produced in the field could induce similar effects as those observed in culture, since bulk measurements of PUA in seawater do not reflect the concentrations in the proximity of PUA-releasing cells in the natural environment. In general, our understanding of PUA release and dynamics at sea is currently very limited, and also, methodologies to detect PUAs at sea are not as refined.

In diatoms, an increase of Dtx is usually related to high light stress and the need to protect the photosynthetic machinery from photoinhibition, Dtx being responsible for the major part of the excess energy dissipation through non-photochemical-quenching (NPQ) ([56] and the references therein). However, the role of the XC in the antioxidant response has been putatively assumed by other authors in response to nutrient limitation [57] or starvation [58], Cd exposure [59] and viral infection [60]. In TW, an increase in Dtx was also observed in reaction to DECA, reflected in a decrease in photosynthetic efficiency [11]. In our experiments, light remained constant, and the increase in Dtx did not couple with variations in NPQ values, indicating that in PUA-exposed SM cultures, the activation of the XC was providing protection against oxidative stress, likely derived from PUA-induced ROS production. Opposite from TW, the photosynthetic efficiency of OCTA-exposed SM cultures was not affected, indicating that the cells were able to cope with PUA maintaining their photosynthetic capability, allowing the cells to efficiently recover after PUA removal, even after 24 h of exposure.

The activation and functioning of the XC is PUA-concentration dependent. Low OCTA concentrations induce a gradual increase of Dtx from 1 h after exposure, parallel to a decrease of Ddx, which was likely converted into Dtx already in the first 20 min. Instead, under the highest OCTA concentration (20 µM), Dtx almost doubled between 20 min and 1 h without any visible Ddx decrease. This indicates that a rapid and strong *de novo* synthesis of both Ddx and Dtx occurred, probably as a protective mechanism against oxidative stress. This XC response has been already observed in *S*. *costatum* following a drastic change in light [61]. Indeed, the decrease of XC pigments during recovery from PUA stress suggests that cells were in a good physiological state and, thus, that the protective mechanism was very effective.

Together with the XC pigments, β-carotene was also involved in the antioxidant defense of SM, as shown by its increase from 3 to 24 h, in the cultures exposed to the highest OCTA concentration. This pigment is an upstream XC pigments precursor [62], as well as an antioxidant molecule, even in marine phytoplankton [60,63,64,65]. Its later increase indicates that it is involved in a later response than the XC [56] and also when oxidative stress is more severe, involving a more intense modification of the membrane apparatus structure.

**Figure 5:**
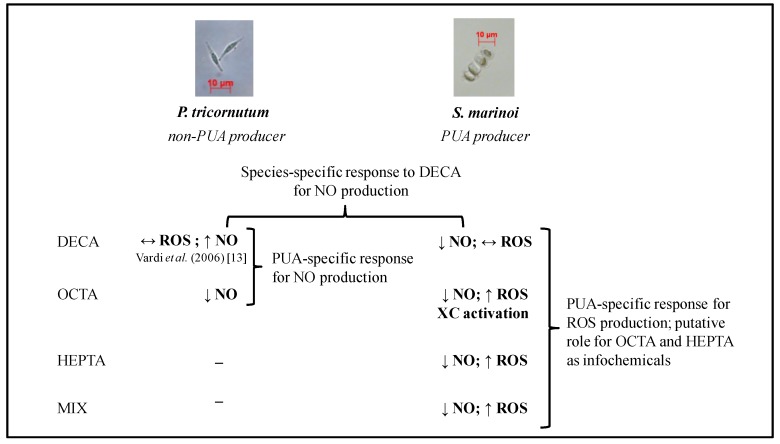
Schematic representation summarizing the results for NO and ROS dynamics in PT and SM (both from this study and available in the literature). PUA: polyunsaturated aldehydes; NO: nitric oxide; ROS: reactive oxygen species; XC: xanthophyll cycle; DECA: 2*E*,4*E*/*Z*-decadienal; OCTA: 2*E*,4*E*/*Z*-octadienal; HEPTA: 2E,4*E*/*Z*-heptadienal; MIX: combination of OCTA and HEPTA. (↑) increase; (↓) decrease; (↔) no change.

A scheme summarizing the different NO and ROS dynamics in PT and SM and the hypotheses proposed in this study is presented in Figure 5.

Finally, it is possible that the green fluorescence detected in SM using DHR was also partly due to peroxynitrite (ONOO^−^) formed by the reaction of NO with an excess of the ROS superoxide anion. This would also be consistent with the observed concomitant decrease of NO and is supported by the strong effect of the ROS scavenger used in our experiments (Tempol), which is also known to attenuate peroxynitrite [66]. Accordingly, preliminary experiments [48] indicate a major involvement of superoxide anion (O_2_^−^) and superoxide dismutase (SOD) activity with consequent H_2_O_2_ formation (and H_2_O_2_-dependent downstream ROS generation) in PUA-exposed SM cells. The data were obtained using two SOD inhibitors, 2ME (2-methoxyestradiol) and DETC (sodium diethyldithiocarbamate trihydrate) and suggest an involvement of the H_2_O_2_-dependent oxidation of DHR123, as previously reported [67,68,69]. Both inhibitors induced a significant decrease of DHR-derived green fluorescence with respect to PUA-treated samples, suggesting that the production of H_2_O_2_ from O_2_^−^ via SOD was prevented. In contrast, the use of the NO scavenger, cPTIO, elicited the opposite response, resulting in an increase of DHR-derived green fluorescence. It has been previously suggested that inhibition of NO synthesis in combination with DHR staining may lead to unexpected results [67]. In fact, by blocking or scavenging NO, the resulting unreacted superoxide can induce a (higher) H_2_O_2_ formation and consequent dye oxidation via a peroxidase- or metal- dependent pathway [67]. However, if superoxide is directed to other cellular targets and, consequently, will not form either ONOO^−^ or H_2_O_2_, then the DHR-derived fluorescence should not change [67]. These preliminary results show that NO scavenging induces an increase in DHR-derived green fluorescence, and it is therefore unlikely that the excess of superoxide is being directed away from the production of H_2_O_2_ and H_2_O_2_-derived products.

### 3.2. Growth

Only 20 µM OCTA (twice the EC_50_ for growth at 24 h) induced total decline of the culture (Table 2), whereas 5 µM and 10 µM inhibited growth in a concentration-dependent way without leading to the complete decline of the culture. When cells were removed from PUA, those that had been exposed to the lower concentrations (5 µM and 10 µM OCTA) were able to resume growth rapidly with faster rates than the control (Table 3). This suggests that they maintained all of their ability to divide and grow, as confirmed by their unaffected photosynthetic efficiency (Table 2). Cultures exposed to the highest OCTA concentration (20 µM) resumed growth at a slower pace and fully recovered only 48 h later (Table 3). This suggests that OCTA concentrations around the EC_50_ for growth at 24 h affect key components of the growth machinery in SM, but not irreversibly, as opposed to TW, which was unable to recover cell division or metabolic activity when exposed to DECA for 24 h, in a threshold- and time-dependent manner [11]. It is also suggested that the blockage in the G1 phase of the cell cycle previously observed in TW [11] might be reversible in SM, probably related to the putative role of PUA as modulators and regulators of population size in this latter species.

## 4. Experimental Section

### 4.1. Culture Conditions and Experimental Design

*Skeletonema marinoi* (SM) (Sarno and Zingone), strain CCMP 2092, and *Phaeodactylum tricornutum* Bohlin (PT), strain CCMP 632, were obtained from the Provasoli-Guillard National Center for Culture of Marine Phytoplankton (Booth Bay Harbor, Maine, USA). Natural filtered seawater (FSW), amended with f/2 nutrients [70], was used as the medium. PT and SM were cultured in a thermostated growth chamber (Hereus Holding GmbH, Hanau, Germany). PT was cultured in polystyrene flasks (Corning Inc., Corning, NY, USA) and SM in 2-L polycarbonate bottles with air bubbling, in order to avoid long chain formation, so as to be amenable to flow cytometry analyses. The axenicity of SM was confirmed before and after every experiment by inoculating 1 mL of culture in a sterile solution of peptone in autoclaved FSW (1 mg·mL^−1^). The cultures were maintained at 20 °C on a 12 h light,12 h dark cycle under a photon flux density of 110 μmol photons·m^−2^·s^−2^. All experiments used exponentially growing cultures, from at least 6 generations, at cell densities from 1 to 2 × 10^5^ cells·mL^−1^ in biological triplicates. Each experiment was performed in triplicates and replicated at least twice. NO and ROS experiments lasted for 180 min, and samples were collected at times 0, 20, 60, 120 and 180 min after inoculation of PUA.

For the recovery experiment, cultures were resuspended in fresh medium without PUA after 24 h of exposure and samples taken after 24 and 48 h for cell counts, XC pigment, NPQ and photosynthetic efficiency analyses.

Cell concentrations were checked by microscopy (Zeiss Axioskop 2, Jena, Germany) from at least 200 cells at 200× magnification using an Axioskop 2 microscope (Carl Zeiss GmbH, Jena, Germany) and Sedgewick-Rafter counting chambers. Growth rates were calculated according to:

μ = ln[(*N*_1_*/N*_0_)/*t*] (1)

where *N*_0_ and *N*_1_ represent cell density at the start and the end of the growth period and *t* is the time between measurements (in days).

The Student’s *t*-test was used to compare average values using Excel software (Microsoft, Redmond, Washington, DC, USA).

### 4.2. PUA

The PUA used in these experiments were: 2*E*,4*E*/*Z*-decadienal (DECA), 2*E*,4*E*/*Z*-octadienal (OCTA), 2*E*,4*E*/*Z*-heptadienal (HEPTA) and a combination of OCTA and HEPTA (MIX) (all from Sigma Aldrich Inc., Milan, Italy). PUA working solutions were prepared by dissolving them in absolute methanol (MeOH) (JT Baker, Phillipsburg, NJ, USA) at RT. The effective PUA concentration of the working solution was assessed spectrophotometrically (Hewlett-Packard 8453, Hewlett-Packard Company, Palo Alto, CA, USA) by using a 274-nm wavelength and a specific molar absorption coefficient of 31,000 [71].

### 4.3. NO and ROS Detection

Staining protocols were modified from [13] for NO detection and from [33] for ROS detection.

Optimal loading times and concentrations were determined empirically for SM. For endogenous NO detection *in vivo*, the fluorescent dye, 4-amino-5-methylamino-2′,7′-difluorofluorescein diacetate (DAF-FM DA), was used. DAF-FM DA is a membrane-permeable ester derivative of DAF-FM. Once inside the cell, the compound DAF-FM DA is first deacetylated by intracellular esterases to become DAF-FM and then further converted into its fluorescent triazole derivative (DAF-FM T) upon reaction with the NO oxidation product, N_2_O_3_ [72]. It has been used on cells from a variety of different organisms, including higher plants, marine invertebrates and phytoplankton [13,20,73,74]. Twenty milliliters of culture were spun and the pellet incubated in the dark in 50 μM DAF-FM DA for 30 min with agitation at 20 °C. Cells were rinsed twice with growth medium and incubated with PUA. DAF-FM DA-green fluorescence was detected by flow cytometry. In order to account for basal levels of NO production, control samples consisted of DAF-treated samples without the addition of PUA, and DAF-fluorescence data of treated samples are therefore represented as increased fluorescence relative to the control. No quenching on NO fluorescence was observed when only MeOH was added to control cultures (treated with DAF), suggesting that the observed decrease in green fluorescence was consistently generated by NO and not due to other artifacts caused by sample manipulation. 

For ROS production, dihydrorhodamine 123 (DHR, Molecular Probes, Leiden, NL, USA) (5 mM stock in DMSO) was used. DHR is oxidized by different reactive oxygen species, including H_2_O_2_ and peroxynitrite (ONOO^−^), to form the fluorescent derivative, rhodamine 123 [67]. DHR has been utilized for the detection of ROS in different cell types [29,33,34]. Samples were incubated with both 10 μM DHR and PUA in the dark at RT. Green fluorescence from the dye was assessed by flow cytometry. Carboxy-PTIO (Vinci-Biochem, Florence, Italy) and Tempol (Sigma-Aldrich Inc., Milan, Italy) were used as scavengers (negative controls) for NO and ROS, respectively, at a final concentration of 100 μM and 5 mM (in FSW). cPTIO is a water-soluble and stable nitric oxide radical scavenger that shows an antagonistic action against NO both in chemical and biological systems via a radical reaction [75], while Tempol is a nitroxide compound, which has been reported to be a general redox cycling agent acting as a catalase (CAT)-like agent [76]. Samples with the scavengers were incubated for 20 min before PUA inoculation. Controls consisted of dye-loaded samples processed like all treatments, except for PUA inoculation.

### 4.4. Flow Cytometry

A Becton-Dickinson Biosciences (Palo Alto, CA, USA) FACScalibur flow cytometer equipped with a 488-nm Ar laser as the excitation source was used. The flow rate was kept constant at 65 μL·min^−1^. Red fluorescence was used as a trigger, with a threshold at Channel 52. Red fluorescence from chlorophylls was collected through a 650 long-pass filter, while green fluorescence from DAF-FM DA and DHR was collected through a 530/30 BP filter. FSW was used as a sheath, and 3.7 μm beads (Coulter Flowset Fluorospheres, Beckman Coulter, Fullerton, CA, USA) were used as internal standards. Data acquisition (10^4^ cells on average for each sample) was performed using CellQuest software (Becton-Dikinson, Palo Alto, CA, USA), while data analysis was performed using FCS4 Express (De Novo Software, Los Angeles, CA, USA). All data are relative units to the beads and are expressed as ratios of control values.

### 4.5. XC Pigments and Photosynthetic Performance

For pigment concentration analyses, 10 mL of culture were filtered through 47-mm GF/F filters (Whatman, Maidstone, UK) and immediately frozen in the dark at −80 °C. Pigments were analyzed by high performance liquid chromatography (HPLC) (Hewlett Packard, series 1100, Kennett Square, PA, USA) shortly after the experiments (1 week at the latest). Filters were extracted in 100% methanol (Sigma-Aldrich, Milan, Italy), and 500 µL of 1 mol·L^−1^ ammonium acetate (final concentration 0.33 mol·L^−1^) were added to the 1-mL pigment extract 5 min before the analysis. A 3-µm C8 BDS column (100 mm × 4.6 mm) was used, and the procedure was the same as described in [77]. The mobile phase was composed of two solvent mixtures: A, methanol/aqueous ammonium acetate, 70:30 vol/vol; and B, methanol. The gradient between the two solvents was programmed as follows: 75% A (0 min), 50% A (1 min), 0% A (15 min), 0% A (18.5 min), 75% A (19 min). Pigments were detected at 440 nm using a photodiode array detector (model DAD series 1100, Hewlett Packard), which gives the 400- to 700-nm spectrum for each detected pigment. A fluorometer (series 1100, Hewlett Packard) allowed the detection of fluorescent pigments, with a 410-nm excitation wavelength and a 665-nm emission wavelength. Identification and quantification of single pigments were realized using chlorophyll (chl) and carotenoid standards obtained from the VKI (Water Quality Institute, Horsholm, Denmark) International Agency for 14C Determination (Horsholm, Denmark).

The photochemical efficiency of photosystem (PS) II was estimated by a Phyto-PAM fluorometer (Heinz Walz GmbH, Effeltrich, Germany). Triplicate variable fluorescence analysis was performed on 15-min dark-acclimated samples, to measure the maximal photochemical efficiency (Fv:Fm, dark-acclimated samples). Fm was measured after a saturating pulse of red light (2400 µmol photons·m^−2^·s^−1^, lasting 450 ms), causing a complete reduction of the PSII acceptor pool.

The non-photochemical quenching of fluorescence (NPQ) was quantified using the Stern–Volmer equation, where Fm and Fm′ are the maximal PSII fluorescence yield for dark-adapted and illuminated cells, respectively:

NPQ = (Fm:Fm′) ‒ 1
(2)

## 5. Conclusions

### Implications and Ecological Hypotheses

It is believed that chemically-mediated interactions have driven the evolution of certain organisms by selecting those individuals that had the ability to either resist, exploit or avoid external metabolites from close-by cells [78]. It is also claimed that this is only expected if chemically-mediated interactions were sporadic, as happens during bloom events [79].

Our data highlight a wide variability of responses to PUA. The type of response depends both on the concentration of PUA to which cells are exposed and the time of exposure. Moreover, different pathways are activated in response to the PUA to which a diatom is exposed, depending also on the diatom species.

From an ecological point of view, our results suggest that diatom species perceive and are able to discriminate the PUA that they are exposed to, probably depending on their adaptive traits. SM is a widespread and bloom-forming marine diatom [43], whereas PT is not very well represented in the natural environment, and it has not been reported to form blooms in nature [80]. SM is a PUA producer, especially of OCTA and HEPTA, which are the most common PUA represented in marine diatoms and found dissolved in seawater after a bloom [49]. On the other hand, PT does not produce any PUA [2]. We therefore suggest that SM recognizes OCTA and HEPTA as intracellular signaling molecules and uses them as infochemicals, while PT reacts to PUA as external deleterious stimuli (probably allelochemicals) [81]. Tests on other diatom species and/or other taxa are needed before concluding that this is a general rule in diatoms and phytoplankton.

We can speculate that the physiological responses documented in this paper are expected to occur at sea during a SM bloom, when nutrients become limiting and senescent cells lyse [82], releasing and increasing the local concentrations of PUA. The response of a PUA-producing diatom to released PUA is modulated by and highly dependent upon the time of exposure and the PUA concentration, both in terms of ROS production and antioxidant defense, leading to increased resistance or death. At intermediate PUA concentrations, cells able to protect themselves are likely to slow down their growth rates, maintaining at the same time their photosynthetic performance. This is expected to allow a quick recovery in case the chemical stress is removed, e.g., by mixing or physical advection or degradation. In this case, the population modulates its size to the new limiting conditions and assures a recovery, increasing its competitiveness as soon as it experiences better environmental conditions.

In conclusion, we suggest that the interactions between chemical signals and reactive pathways underlie the functional diversity of species and their ability to cope with the environment. Indeed, the physiological responses to stimuli and biological interactions are intertangled and can shape ecosystems in a dynamical way, determining the ecological success of a species and its role.
